# An Adult Patient with Ocular Myasthenia and Unusually Long Spontaneous Remission

**DOI:** 10.1155/2014/372769

**Published:** 2014-04-13

**Authors:** Jasem Al-Hashel, Hanaa M. Rashad, Rossen T. Rousseff

**Affiliations:** Department of Neurology, Ibn Sina Hospital, P.O. Box 25427, 13115 Safat, Kuwait

## Abstract

A male patient developed ocular myasthenia gravis (MG) at the age of 33. He was anti-acetylcholine receptor antibody (anti-AChR Ab) negative. He received cholinesterase blocker for 5 months and went into a complete clinical remission that lasted untreated for 17 years. He relapsed recently with ocular symptoms only. He is now anti-AChR Ab positive and SFEMG is abnormal in a facial muscle. The patient is controlled with steroids. He had one of the longest spontaneous remissions reported in the natural history of MG, particularly unusual for an adult with the disease.

## 1. Introduction

The natural history of myasthenia gravis (MG) in untreated patients is mostly derived from historical cohorts [1, 2]. Spontaneous remissions without treatment are common in the first year after onset, but they rarely last long [3]. They are more common in juvenile MG [4]. A remission in ocular MG under continuing anticholinesterase treatment is expected in up to 50% of cases, but it mostly represents symptom control rather than true cessation of disease activity [5].

We observed a patient with ocular MG who became disease-free after a brief period of anticholinesterase treatment and relapsed after 17 years.

## 2. Case Presentation

A male patient presented in March 1995 with double vision in all directions and drooping of the left eyelid. These started 3 months earlier, tended to increase towards the evening, and fluctuated over consecutive days. The diplopia impeded seriously his work as a heavy excavator driver.

The patient's medical and family history was unremarkable. General examination was normal. Neurological examination disclosed fatiguable ptosis of both upper lids, diplopia in all directions, and weakness of the extraocular muscles. The latter varied in distribution over consecutive examinations but mostly involved the adduction in both eyes and did not follow cranial nerve(s) pattern. The rest of the neurological status was normal; in particular facial muscles fatigability or weakness and problems with chewing, swallowing, or speech were not reported or noticed. The ice pack test was positive. Laboratory results including anti-AChR Ab and thyroid function tests were in the normal range. Repetitive nerve stimulation (RNS) in the nasalis and abductor digiti minimi muscles showed no decrement. Orbital CT was normal, while chest CT excluded thymus pathology.

The patient was started on pyridostigmine bromide 60 mg every six hours, which was well tolerated and completely abolished his symptoms. In 5 months he developed a complete remission, stopped the medication on his own, and did not seek medical help over 17 years.

In May 2013 the patient's symptoms and signs relapsed in a pattern very similar to his initial presentation. He sought medical advice and was found again to have diplopia in all directions, a left eyelid fatigable ptosis, and extraocular muscle weakness in fluctuating pattern. It affected mostly the adduction of both eyes, abduction and elevation on the right, and abduction on the left. His general examination and neurological status were again normal except for the ocular motor disturbances. Routine laboratory studies and anti-AChR Ab and anti-MuSK antibodies were negative. RNS was unremarkable in the nasalis, trapezoid, and abductor digiti minimi muscles. A SFEMG of the frontalis muscle one month after onset was normal. However the follow-up study at 2 months (volitional jitter in the orbicularis oculi muscle, concentric needle, 20 fibers) was definitely abnormal with 2 blocking fibers, 7 fibers with increased jitter, and abnormal mean MCD per study. SFEMG of the extensor digitorum muscle (volitional, concentric needle) was normal. Orbital and chest CT was unremarkable. A repeated anti-AChR Ab testing returned positive. The patient is now treated with prednisolone and his myasthenia is controlled with complete clinical remission.

## 3. Discussion

We present a patient with ocular myasthenia who developed a true remission after a relatively short exposure to cholinesterase blockers only. The remission lasted about 17 years and is among the longest reported in literature.

The natural course of untreated MG is less clear and is mostly derived from the earlier studies, predating the use of thymectomy and immunosuppression [1–3]. Spontaneous remissions are common in 11 to 21% of patients and tend to occur in the early period, but they rarely last more than few months, exceptionally reaching several years [2, 3]. They are an important phenomenon, as the assessment of medication effect or thymectomy in early MG must take into account the possibility of spontaneous remission compounding the results [6]. The criteria for remission are not settled. Some authors accept as a true remission a one-year disease-free period while others suggested that the ability of the patient to return to his everyday activities is sufficient to accept remission [3, 7]. This might explain the discrepancies in the reported duration in historical cohorts. Besides, persisting disease activity has been documented by immunological and/or SFEMG abnormalities in patients with apparent clinical remission either under or without treatment [8, 9].

Treatment with cholinesterase inhibitors is purely symptomatic and does not suppress the underlying autoimmune pathology [10]. In fact, such agents may aggravate the immune conflict via their effect on the acetylcholinesterase expression [11]. Complex immunogenetical mechanisms may produce frank myopathy under anticholinesterase treatment [12]. Anti-AChE therapy alone is not expected to result in remission; the latter may be expected only with thymectomy or immunosuppression.

Few well documented cases of long-lasting spontaneous remissions are found in literature. An early article by Schwab 1953 mentions a 17-year disease-free period in a patient; it is unclear whether she/he relapsed [1]. Oosterhuis reports two untreated patients with ocular myasthenia with a 6-year long remission [2]. Three cases of ocular myasthenia in childhood with spontaneous remission lasting from 4 to 14 years are better documented [13]. Prepubertal children with ocular myasthenia are more prone to spontaneous remission than adults [14]. Recently we find the report of an 8-year-old boy presenting with generalized MG who recovered within 1 month without any immunomodulation. His spontaneous remission lasted 6 years and was ended by a relapse with bulbar symptoms [15].

Such phenomena pose some interesting questions regarding the pathophysiology of MG. In experimental autoimmune MG repeated inoculations with AChE receptor are necessary to produce chronic progressive MG and spontaneous remissions occur in different percent of animals according to the number of inoculations, amount of immunogen, and other factors [16, 17]. Could rare human MG cases with very long spontaneous remission parallel a self-limited “first inoculation” response in experimental myasthenia? In our patient, the AChR Ab were negative during the brief initial episode and early in the relapse, but “seroconversion” followed.

As discussed in detail elsewhere [18] transient anxiety may play a role in the fluctuating course of MG via its effect on the cholinesterase activity. Assessment of serum cholinesterase levels in this patient was unfortunately not done during the initial episode; however, it should be considered in further studies on disease course in MG.

Most of the reported patients with long spontaneous remission were children and had purely ocular manifestations. This may imply some immunological or biological trait in a heterogeneous disorder like MG [19] or simply reflect the early and preferential involvement of the extraocular muscles that results from a complex of factors [20].

In summary, our observation turns attention to a rare variant of the natural course of untreated MG which is particularly unusual patients with adult onset of the disease.

## References

[1] Schwab RS, Leland CC (1953). Sex and age in myasthenia gravis as critical factors in incidence and remission (spontaneous and following thymectomy). *Journal of the American Medical Association*.

[2] Grob D, Brunner NG, Namba T (1981). The natural course of myasthenia gravis and effect of therapeutic measures. *Annals of the New York Academy of Sciences*.

[3] Oosterhuis HJGH (1989). The natural course of myasthenia gravis: a long term follow up study. *Journal of Neurology Neurosurgery and Psychiatry*.

[4] Rodriguez M, Gomez MR, Howard FM, Taylor WF (1983). Myasthenia gravis in children: long-term follow-up. *Annals of Neurology*.

[5] Kupersmith MJ (2009). Ocular myasthenia gravis: treatment successes and failures in patients with long-term follow-up. *Journal of Neurology*.

[6] Kaminski HJ, Daroff RB (2000). Treatment of ocular myasthenia: steroids only when compelled. *Archives of Neurology*.

[7] Simpson JA (1958). An evaluation of thymectomy in myasthenia gravis. *Brain*.

[8] Mano K, Takegami T, Okamoto S, Takahashi A (1993). Remission of myasthenia gravis: clinical, electrophysiological and immunological studies. *Nagoya Journal of Medical Science*.

[9] Kostera-Pruszczyk A, Emeryk-Szajewska B, Witalska J (2002). Clinical, electrophysiological and immunological remissions after thymectomy in myasthenia gravis. *Clinical Neurophysiology*.

[10] Juel VC, Massey JM (2007). Myasthenia gravis. *Orphanet Journal of Rare Diseases*.

[11] Brenner T, Hamra-Amitay Y, Evron T, Boneva N, Seidman S, Soreq H (2003). The role of readthrough acetylcholinesterase in the pathophysiology of myasthenia gravis. *The FASEB Journal*.

[12] Lev-Lehman E, Evron T, Broide RS (2000). Synaptogenesis and myopathy under acetylcholinesterase overexpression. *Journal of Molecular Neuroscience*.

[13] Rollinson RD, Fenichel GM (1981). Relapsing ocular myasthenia. *Neurology*.

[14] Ortiz S, Borchert M (2008). Long-term outcomes of pediatric ocular myasthenia gravis. *Ophthalmology*.

[15] Anlar B, Yilmaz V, Saruhan-Direskeneli G (2009). Long remission in muscle-specific kinase antibody-positive juvenile myasthenia. *Pediatric Neurology*.

[16] Lennon VA, Lindstrom JM, Seybold ME (1975). Experimental autoimmune myasthenia: a model of myasthenia gravis in rats and guinea pigs. *Journal of Experimental Medicine*.

[17] Wu B, Goluszko E, Huda R, Tüzün E, Christadoss P (2011). Experimental autoimmune myasthenia gravis in the mouse. *Current Protocols in Immunology*.

[18] Shenhar-Tsarfaty S, Berliner S, Bornstein NM, Soreq H (2013). Cholinesterases as biomarkers for parasympathetic dysfunction and inflammation-related disease. *Journal of Molecular Neuroscience*.

[19] Meriggioli MN, Sanders DB (2009). Autoimmune myasthenia gravis: emerging clinical and biological heterogeneity. *The Lancet Neurology*.

[20] Serra A, Ruff RL, Leigh RJ (2012). Neuromuscular transmission failure in myasthenia gravis: decrement of safety factor and susceptibility of extraocular muscles. *Annals of the New York Academy of Sciences*.

